# T-DM1, a novel antibody–drug conjugate, is highly effective against primary HER2 overexpressing uterine serous carcinoma in vitro and in vivo

**DOI:** 10.1002/cam4.274

**Published:** 2014-06-02

**Authors:** Diana P English, Stefania Bellone, Carlton L Schwab, Ileana Bortolomai, Elena Bonazzoli, Emiliano Cocco, Natalia Buza, Pei Hui, Salvatore Lopez, Elena Ratner, Dan-Arin Silasi, Masoud Azodi, Peter E Schwartz, Thomas J Rutherford, Alessandro D Santin

**Affiliations:** 1 Division of Gynecologic Oncology, Department of Obstetrics, Gynecology and Reproductive Sciences, Yale University School of MedicineConnecticut, 06520; 2 Department of Pathology, Yale University School of MedicineConnecticut, 06520; 3 Division of Gynecologic Oncology, University Campus Biomedico of RomeRome, Italy

**Keywords:** Ado-trastuzumab emtansine, HER2, T-DM1, trastuzumab, uterine serous carcinoma

## Abstract

Amplification of *c-erbB2* has been reported in over 30% of uterine serous carcinoma (USC) and found to confer poor survival because of high proliferation and increased resistance to therapy. In this study, we evaluated for the first time Trastuzumab emtansine (T-DM1), a novel antibody–drug conjugate, against multiple epidermal growth factor receptor-2 (HER2)-positive USC cells in vitro followed by developing a supportive in vivo model. Fifteen primary USC cell lines were assessed by immunohistochemistry (IHC) and flow cytometry for HER2 protein expression. *C-erbB2* gene amplification was evaluated using fluorescent in situ hybridization. Sensitivity to T-DM1 and trastuzumab (T)-induced antibody-dependent cell-mediated cytotoxicity was evaluated in 5-h chromium release assays. T-DM1 and T cytostatic and apoptotic activities were evaluated using flow-cytometry-based proliferation assays. In vivo activity of T-DM1 versus T in USC xenografts in SCID mice was also evaluated. High levels of HER2 protein overexpression and HER2 gene amplification were detected in 33% of USC cell lines. T-DM1 was considerably more effective than trastuzumab in inhibiting cell proliferation and in causing apoptosis (*P* = 0.004) of USC showing HER2 overexpression. Importantly, T-DM1 was highly active at reducing tumor formation in vivo in USC xenografts overexpressing HER2 (*P* = 0.04) and mice treated with TDM-1 had significantly longer survival when compared to T-treated mice and control mice (*P* ≤ 0.0001). T-DM1 shows promising antitumor effect in HER2-positive USC cell lines and USC xenografts and its activity is significantly higher when compared to T. T-DM1 may represent a novel treatment option for HER2-positive USC patients with disease refractory to trastuzumab and traditional chemotherapy.

## Introduction

Trastuzumab emtansine (T-DM1, Genentech/Roche, South San Francisco, CA) is a novel antibody–drug conjugate that comprised trastuzumab covalently linked to the antimicrotubule agent DM1 1. Trastuzumab is a recombinant humanized monoclonal antibody against the extracellular domain of epidermal growth factor receptor-2 (HER2) and is currently approved for the treatment of both metastatic and early-stage breast cancer as well as gastric cancer overexpressing HER2 2. DM1 belongs to the maytansine class of chemotherapeutic agents. On average, 3–4 molecules of DM1 are conjugated to each trastuzumab molecule. T-DM1 is an agent aimed at delivering the highly potent DM1 into HER2 overexpressing cells via receptor-mediated endocytosis 1. In one pivotal trial (EMILIA), T-DM1 demonstrated robust clinical activity in patients with trastuzumab-refractory HER2-positive breast cancer with a 43.6% objective response rate and median progression-free survival of 9.6 months 3.

Uterine serous carcinoma (USC) accounts for ∼10% of endometrial cancer. This subtype of endometrial cancer is highly biologically aggressive and accounts for a disproportionately large number of endometrial cancer deaths 4,5. There is such a poor prognosis associated with this subtype of endometrial cancer that alternative treatment approaches are routinely being investigated. Histologically, USC and high-grade ovarian serous tumors are indistinguishable. Molecular profiling studies have demonstrated HER2 to be one of the most overexpressed genes in USC and may be used to distinguish USC from ovarian serous tumors 6. HER2 is a member of the epidermal growth factor receptor (EGFR) family of receptor tyrosine kinases. HER2 overexpression has been reported to range from 18% to 80% in USC depending on the immunohistochemistry (IHC) technique used 7.

Although the mechanisms underlying trastuzumab's clinical benefit have not been completely elucidated, its therapeutic benefit is attributed to disruption of signaling through the PI3K/AKT/mTOR pathway in HER2 overexpressed malignant cells as well as through the recruitment of NK cells and initiation of antibody-dependent cell-mediated cytotoxicity (ADCC) 8. T-DM1 retains the above-mentioned clinical benefit of trastuzumab and is designed to deliver the intracellular cytotoxic effects of DM1 into the HER2 overexpressing tumor cells. Up to now, no previous experiences with T-DM1 against HER2-positive USC cells in vitro or against USC xenografts have been reported in the English literature.

## Materials and Methods

### Establishment of USC cell lines

Study approval was obtained from the Institutional Review Board, and all patients signed consent prior to tissue collection according to the institutional guidelines. A total of 15 USC cell lines were established after the sterile processing of fresh tumor biopsy samples, as described previously 9. All USC cell lines were established from biopsies taken from chemotherapy-naïve patients at the time of primary staging surgery (Table 1). Tumors were staged according to the International Federation of Gynecology and Obstetrics staging system. Patient characteristics are noted in Table 1. Primary USC cell lines with limited passages (i.e., <50) were analyzed by flow cytometry for HER2 expression both immediately after tumor processing as well as after being cultured in vitro for 1 week to 5 years. This was evaluated with Trastuzumab (Herceptin; Genentech, San Francisco, CA) as a humanized monoclonal antibody (mAb) of the IgG1 isotype and is further explained under the flow cytometry section of the Materials and Methods. The corresponding cell blocks were also analyzed for HER2 surface expression by IHC and *c-erbB-2* gene amplification by fluorescent in situ hybridization (FISH).

**Table 1:** Patient characteristics

Patient	Age (years)	Race	FIGO stage	USPC histology
USC ARK-1	62	AA	IVA	Pure
USC ARK-2	63	AA	IVB	Pure
USC ARK-3	59	AA	IVB	Mixed
USC ARK-4	73	C	IVB	Pure
USC ARK-5	73	AA	IIIC	Pure
USC ARK-6	62	C	IB	Mixed
USC ARK-7	75	C	IIC	Pure
USC ARK-8	88	C	IIIA	Pure
USC ARK-9	73	AA	IIIC	Mixed
USC ARK-11	80	AA	IIIC	Mixed
USC ARK-12	64	AA	IVB	Pure
USC ARK-13	67	C	IVB	Pure
USC ARK-14	73	AA	IV	Pure
USC ARK-19	65	C	IA	Pure
USC ARK-21	70	C	IA	Pure

AA, African-American; C, Caucasian; FIGO, International Federation of Gynecology and Obstetrics stage 1988.

### Immunostaining of cell blocks of primary USC

Cell blocks were obtained from all 15 USC cell lines and reviewed by a gynecologic surgical pathologist to confirm the presence of serous carcinoma cells. The level of HER2 expression was then evaluated as described previously 9. Briefly, HER2 immunohistochemical staining was performed on paraffin-embedded 5-*μ*m sections of cell blocks after deparaffinization and rehydration, using the *c-erbB-2* antibody (Thermo Fisher Scientific, Fremont, CA) at 1:800 dilution. HER2 staining intensity was graded per the American Society of Clinical Oncology and the College of American Pathologists (ASCO/CAP) 2007 breast scoring criteria.

### FISH of cell blocks from primary USC

Fluorescent in situ hybridization analysis was performed using the PathVysion HER2 DNA FISH Kit (Abbott Molecular Inc., Abbott Park, IL) according to the manufacturer's instructions. Cell block sections of 5 *μ*m were deparaffinized and rehydrated, followed by acid pretreatment and proteinase K digestion. A probe mix containing an orange probe directed against the *c-erbB-2* gene (Vysis, Inc., Downers Grove, IL, LSI HER2) and a green probe directed against the pericentromeric region of chromosome 17 (Vysis CEP 17) were added and specimens were denatured for 5 min at 73°C. Slides were then incubated overnight in a humidity chamber at 37°C and washed the day after when a fluorescence mounting medium, containing 4, 6-diamidino-2-phenylindole (DAPI), was applied. Fluorescent signals in at least 30 nonoverlapping interphase nuclei with intact morphology were scored using a Zeiss Axioplan 2 microscope (Carl Zeiss Meditec, Inc., Dublin, CA) with a 100× planar objective, using a triple band-pass filter that permits simultaneous blue, green, and red colors. A case was scored as amplified when the ratio of the number of fluorescent signals of *c-erbB-2* gene to chromosome 17 was ≥2.

### Quantitative real-time polymerase chain reaction

RNA isolation from all 15 USC cell lines and from normal endometrium cell controls used in these experiments was performed using TRIzol Reagent (Invitrogen, Carlsbad, CA) according to the manufacturer's instructions. Quantitative PCR was carried out to evaluate the expression level of HER2 in all samples with a 7500 real-time PCR system using the recommended protocol by the manufacturer (Assay ID: Hs00170433_m1; Applied Biosystems, Foster City, CA). Each reaction was run in duplicate. The internal control, glyceraldehyde-3-phosphate dehydrogenase Assay-on-Demand Hs99999905_ml (Applied Biosystems), was used to normalize variations in cDNA quantities from different samples. The comparative threshold cycle (C_T_) method was used for the calculation of amplification fold as specified by the manufacturer.

### Flow cytometry

Trastuzumab (Herceptin; Genentech) is a humanized mAb of the IgG1 isotype that binds with high affinity to the extracellular domain of the HER2 receptor. The USC cell lines were incubated with 2.5 *μ*g/mL of trastuzumab for 30 min on ice. Rituximab, chimeric anti-CD20 mAb (Rituxan; Genentech) 5 *μ*g/mL was used as a negative control. For staining, a fluorescein isothiocyanate-conjugated goat anti-human F(ab1)2 immunoglobulin was used as a secondary reagent (BioSource International, Camarillo, CA). Analysis was conducted with a FACScalibur, using Cell Quest software (BD Biosciences, San Diego, CA). Cell cycle position was identified by the DNA content of cells as determined by propidium iodide (PI) staining. For each analysis 10,000 gated events were collected to permit cell cycle analysis. Data analysis was performed using Cell Quest (BD Biosciences) and FlowJo v8.8.2 (TreeStar Inc., Ashland, OR)

### Tests for ADCC

Standard 5-h chromium (^51^Cr) release assay was performed to measure the cytotoxic reactivity of Ficoll-Hypaque-separated peripheral blood lymphocytes (PBLs) from several healthy donors in combination with trastuzumab or T-DM1 against the 15 USC target cell lines at effector to target ratios (E:T) of 15:1 and 30:1. The release of ^51^Cr from target cells was measured as evidence of tumor cell lysis after exposure of the tumor cells to 2.5 *μ*g/mL of trastuzumab or 2.5 *μ*g/mL of T-DM1. Dose–response experiments were performed in order to determine the optimal antibody dosing for ADCC experiments. Chimeric anti-CD20 mAb rituximab 2.5 *μ*g/mL was used as the negative control for trastuzumab and T-DM1 in all bioassays. The percentage cytotoxicity of trastuzumab or T-DM1 was calculated by the following formula: % cytotoxicity *=* 100 *×* (*E − S*)/(*T − S*), where *E* is the experimental release, *S* is the spontaneous release by target cells, *T* is the maximum release by target cells lysed with 0.1% SDS.

### Proliferation assay

To evaluate the potential cell cycle and apoptotic effects of T-DM1 versus Trastuzumab on USC cell lines, cells were seeded at log phase of growth in a six-well plate at a density of 50,000–100,000 cells in appropriate culture media. After 24 h, either trastuzumab, rituximab, or T-DM1 was added to a well of final volume of 2 mL, so that the concentration of trastuzumab, rituximab, or T-DM1 was 20 *μ*g/mL, 20 *μ*g/mL, or 2 *μ*g/mL, respectively, in the respective wells. The concentration of rituximab, trastuzumab, and T-DM1 used in the assays were largely determined by dose–response experiments conducted previously for the respective bioassays. After 48–72 h, the trastuzumab, rituximab, and T-DM1 cell cycle and apoptotic activity were evaluated using flow-cytometry-based assays after harvesting the entire well contents, centrifuging and staining with 2 *μ*L of PI as described previously 9.

### In vivo assay of drug effect

Five- to 8-week-old female SCID mice (Harlan Netherlands, Horst, Netherlands) were given a single intraperitoneal (i.p.) injection of 7.5 × 10 USC ARK-2 cells (high HER2 expressor that does not harbor a PIK3CA mutation) in ∼400 *μ*L phosphate-buffered saline as described previously 10. Thereafter, the mice were divided into three treatment groups, namely, rituximab (15 mg/kg), trastuzumab (15 mg/kg), and T-DM1 (15 mg/kg), respectively. Each group consisted of five mice and injections with the treatment drug were administered after a 7-day period was allowed for tumor establishment. All treatment drugs were given as i.p. injections once per week. No signs of general toxicity were seen in any of the three treatment groups. The mice in all three treatment groups were given series of five injections after which they were placed in follow-up and observed for overall survival as the primary outcome measure.

### Statistical analysis

For quantitative real-time PCR (qRT-PCR) data, right skewing was removed by taking copy number ratios relative to the lowest expressing normal endometrium (relative copy number), and then log_2_ transforming them to ΔC_T_ values. Group means with 95% confidence intervals (CI) were then calculated for the relative copy numbers. Differences in HER2 expression by flow cytometry were analyzed by the unpaired *t*-test, and a *P* < 0.05 among the samples was considered to be significant. The Wilcoxon rank-sum test was used to compare USC cell lines to normal endometrium for differences in IHC staining intensity. The unpaired *t*-test was used to evaluate the differences in ADCC levels by 5-h chromium release assays and the inhibition of proliferation in the USC cell lines after exposure to trastuzumab versus T-DM1. Overall survival data were analyzed and plotted using the Kaplan–Meier method. Survival curves were compared using the log-rank test. Statistical analysis was performed using GraphPad Prism (La Jolla, CA) version 6. A *P* < 0.05 was considered as the level of statistical significance.

## Results

### HER2 expression by IHC in USC cell blocks

**Figure 1:**
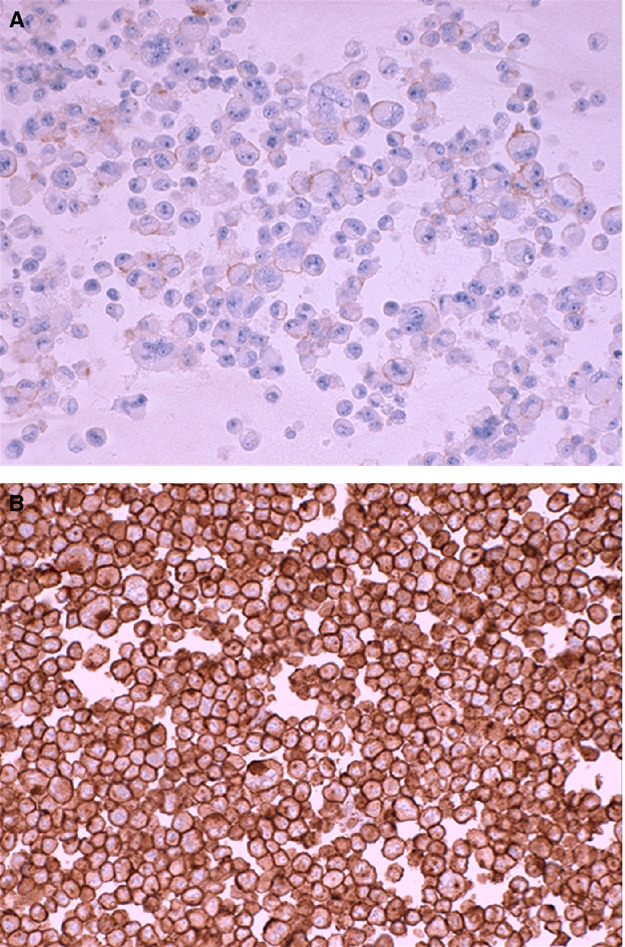
Representative epidermal growth factor receptor-2 (HER2) expression by immunohistochemistry (IHC) in uterine serous carcinoma (USC) cell blocks. USC ARK-6 (A) shows 1+/weak staining for HER2 while USC ARK-1 (B) shows strong membranous (3+) staining for HER2.

High levels of HER2 protein expression by immunohistochemistry (+3 staining) were detected in 33% of the USC cell lines (i.e., 5 of 15). Three cell lines had no HER2 expression on IHC, whereas the other seven cell lines had +1/+2 expression (Table 2). The HER2 expression by IHC in the high expressor group was significantly different compared to normal endometrial tissue (data not shown). Figure1 shows representative HER2 expression by IHC in low HER2-expressing cell line (USC ARK-6) and high HER2-expressing cell line (USC ARK-1) cell blocks.

**Table 2:** Immunohistochemistry for HER2 on cell and tissue blocks and *c-erbB2* gene amplification in primary USC cell lines

Sample	IHC cell block	FISH	IHC tissue BLOCK	PI3KCA mutations
USC ARK-1	3+	Amplified	3+	Detected
USC ARK-2	3+	Amplified	3+	Not detected
USC ARK-3	3+	Amplified	3+	Not detected
USC ARK-4	0	Not amplified	1+	Not detected
USC ARK-5	0	Not amplified	1+	Not detected
USC ARK-6	1+	Not amplified	1+	Not detected
USC ARK-7	2+	Not amplified	2+	Not detected
USC ARK-8	1+	Not amplified	1+	Not detected
USC ARK-9	3+	Amplified	3+	Detected
USC ARK-11	1+	Not amplified	1+	Not detected
USC ARK-12	0	Not amplified	0	Not detected
USC ARK-13	2+	Not amplified	2+	Not detected
USC ARK-14	2+	Not amplified	1+	Not detected
USC ARK-19	1+	Not amplified	1+	Not detected
USC ARK-21	3+	Amplified	3+	Not detected

FISH, fluorescent in situ hybridization; HER2, epidermal growth factor receptor-2; IHC, immunohistochemistry; USC, uterine serous carcinoma.

### HER2 expression by qRT-PCR in USC

Uterine serous adenocarcinoma cell lines grown as primary cultures were tested by qRT-PCR to confirm the different HER2 surface receptor expressions at the mRNA level. Negative controls were normal endometrial samples. The HER2 mRNA level in the high expressor group was significantly different compared to normal endometrial tissue (data not shown). The mRNA copy number for USC ARK-1, USC ARK-2, USC ARK-3, USC ARK-9, and USC ARK-21 (high expressors) were 2749.6, 4478.9, 4993.8, 4323.6, and 750.4, respectively, with a group mean mRNA copy number of 3459 and 95% CI; 1310–5606. This was compared with mRNA copy numbers of between 0.02 and 117.2 for USC ARK-4, USC ARK-5, USC ARK-6, USC ARK-7, USC ARK-8, USC ARK-11, USC ARK-12, USC ARK-13, USC ARK-14, USC ARK-19 (low expressors) with a group mean and 95% CI of 45.34; 15.44–75.24; *P* < 0.0001; (Table 3). These data are in agreement with the results obtained by IHC as shown in Table 2.

**Table 3:** USC cell lines HER2 mRNA copy number by qRT-PCR and mean fluorescence intensity data

CODE	Detector	mRNA copy number	Her2/neu IHC	Mean fluorescence intensity
USC ARK-1	HER2/Neu	2749.585	3+	339.5
USC ARK-2	HER2/Neu	4478.949	3+	710.3
USC ARK-3	HER2/Neu	4993.806	3+	228.7
USC ARK-4	HER2/Neu	52.869	1+	10.8
USC ARK-5	HER2/Neu	95.351	1+	18.7
USC ARK-6	HER2/Neu	44.319	1+	13.0
USC ARK-7	HER2/Neu	45.694	2+	47.4
USC ARK-8	HER2/Neu	17.671	1+	24.8
USC ARK-9	HER2/Neu	4323.614	3+	285.7
USC ARK-11	HER2/Neu	117.229	1+	42.6
USC ARK-12	HER2/Neu	0.898	0	32.8
USC ARK-13	HER2/Neu	0.018	1+	8.8
USC ARK-14	HER2/Neu	0.068	2+	26.6
USC ARK-19	HER2/Neu	79.274	1+	57.3
USC ARK-21	HER2/Neu	750.426	3+	159.3
CALIBRATOR	HER2/Neu	1		

### *erbB-2* gene amplification by FISH

Fluorescent in situ hybridization was performed on cell blocks obtained from all 15 primary cell lines used in the cytotoxicity experiments. Tests results are shown in Table 2. *c-erbB-2* gene amplification was detected in USC ARK-1, USC ARK-2, USC ARK-3, USC ARK-9, and USC ARK-21, thus supporting the premise that strong receptor expression and high HER2 mRNA levels were likely as a result of *c-erbB-2* gene amplification. In contrast, USC ARK-4, USC ARK-5, USC ARK-6, USC ARK-7, USC ARK-8, USC ARK-11, USC ARK-12, USC ARK-13, USC ARK-14, and USC ARK-19 were found to be negative for *c-erbB-2* gene amplification (Table 2).

### HER2 expression by flow cytometry

**Figure 2:**
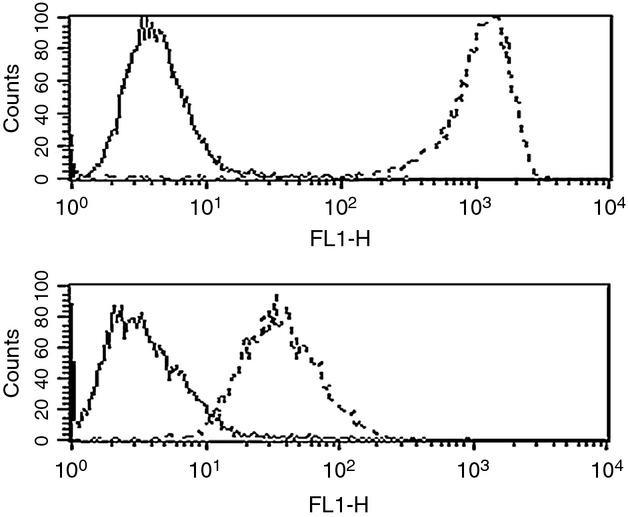
Flow cytometry histograms of primary uterine serous carcinoma (USC) cell lines showing high epidermal growth factor receptor-2 (HER2)-expressing cell line (USC ARK-1) above and low HER2-expressing line (USC ARK-4) below. Rituximab anti-CD20 control antibody is the solid line and trastuzumab is the dashed line. High HER2-expressing cell line shows significantly higher mean fluorescence intensity with trastuzumab versus low HER2-expressing cell line.

HER2 receptor expression was evaluated by FACS analysis on all 15 primary USC cell lines using trastuzumab. Cell lines with low HER2 protein expression by qRT-PCR were used as negative controls. As representatively shown in Figure2 very high reactivity against HER2 was found in 100% of primary USC ARK-1, USC ARK-2, USC ARK-3, USC ARK-9, and USC ARK-21 cell lines using trastuzumab (Table 3). In this group, the mean fluorescence intensity (MFI) ranged from 159.3 to 710.3 (Table 3). Significantly lower HER2 expression was observed in USC ARK-4, USC ARK-5, USC ARK-6, USC ARK-8, USC ARK-11, USC ARK-12, USC ARK-13, USC ARK-14, and USC ARK-19 with a MFI range 8.8–57.3 (Table 3). Figure2 shows two examples of flow cytometry histograms of primary USC cell lines showing high HER2-expressing cell line (USC ARK-1) above and low HER2-expressing line (USC ARK-4) below.

### T-DM1 and trastuzumab mediated ADCC against HER2-positive primary USC

All 15 primary USC cell lines were tested for their sensitivity to PBL-mediated cytotoxicity when challenged with heterologous PBLs collected from several healthy donors in a standard 5-h ^51^Cr release assay. USC cells were consistently found to be resistant to PBL-mediated cytotoxicity when combined with PBLs and rituximab (2.5 *μ*g/mL) at E:T ratios of 15:1 and 30:1 (mean ± SEM cytotoxicity of 1.95 ± 1.04% with PBL alone and mean ± SEM cytotoxicity of 1.22 ± 0.96% in the presence of rituximab + PBL, respectively).

**Figure 3:**
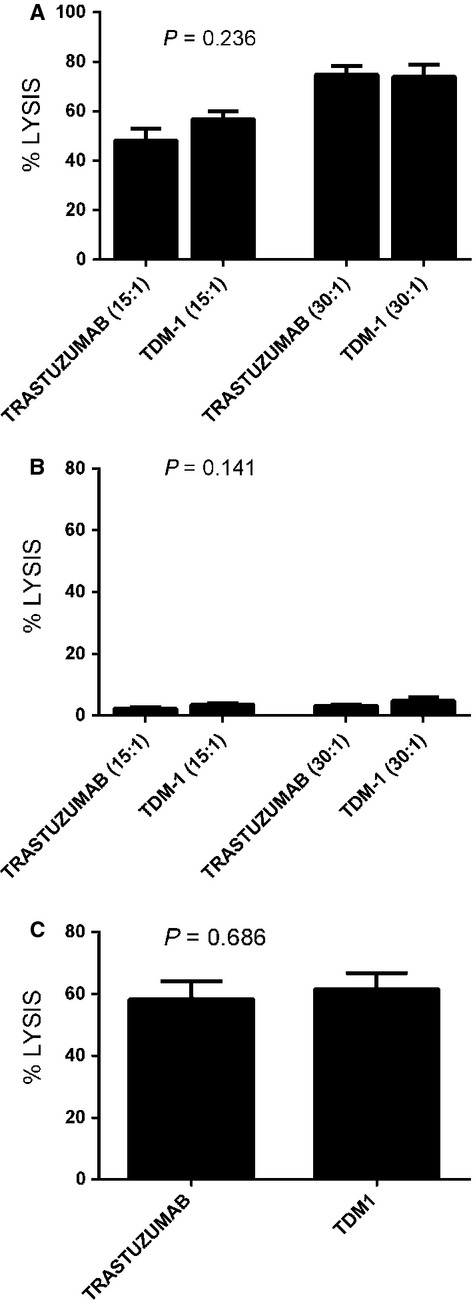
A representative antibody-dependent cell-mediated cytotoxicity (ADCC) with trastuzumab versus T-DM1 at two different effectors to target ratios in a uterine serous carcinoma (USC) cell line with high epidermal growth factor receptor-2 (HER2 expression (USC ARK-2, (A) versus a low HER2-expressing cell line (USC ARK-4, (B) is shown. In (C), ADCC of T versus T-DM1 (mean ± SD) in the five available USC cell lines with high HER2 expression (USC ARK-1, 2, 3, 9, and 21). No significant difference in ADCC is seen with T versus T-DM1 (*P* = 0.686).

We then investigated the sensitivity of USC cell lines to heterologous PBLs in the presence of trastuzumab 2.5 *μ*g/mL and T-DM1 at 2.5 *μ*g/mL. T-DM1 and trastuzumab (T) were similarly effective in inducing strong ADCC against all primary USC cell lines expressing high levels of HER2 (i.e., USC ARK-1, USC ARK-2, USC ARK-3, USC ARK-9, and USC ARK-21) with mean cytotoxicity ± SEM, 61.6 ± 5.3% versus 58.4 ± 5.74%, TDM-1 versus T, *P* = 0.686 (Fig.3), while negligible ADCC was detected against USC cell lines with low/negative HER2 expression when combined with PBLs and trastuzumab or T-DM1 compared to control (target cells alone or PBLs and rituximab) in multiple experiments (Fig.3). Indeed, in cell lines expressing low levels of HER2, the cytotoxicity achieved with trastuzumab or T-DM1 was on the order of mean ± SEM (3.02 ± 0.98, range 0.23–4.90) compared with PBLs and rituximab; mean ± SEM (0.83 ± 0.03, range 0.79–0.86).

### T-DM1 is more effective than trastuzumab at inhibiting USC cell proliferation

**Figure 4:**
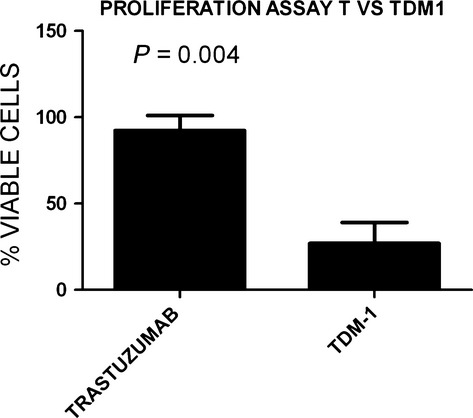
T versus T-DM1 proliferation assay results (mean ± SD) for all five high HER2 overexpressing cell lines. T-DM1 significantly inhibited USC proliferation compared to T (*P* = 0.004).

We subsequently examined sensitivity of the USC cell lines to both trastuzumab and T-DM1. T-DM1 was dramatically more effective than T in inhibiting HER2 overexpressing USC cell proliferation and in causing apoptosis in these cell lines (Fig.4). T-DM1 showed significant activity against all USC cell lines including cell lines endowed with primary resistance to T (i.e., USC ARK-1, USC ARK-2, USC ARK-3, and USC ARK-9). The mean number of viable cells ± SEM was 26.9 ± 12.2% versus 92.4 ± 8.8% for T-DM1 versus trastuzumab, respectively, (*P* = 0.004; Fig.4). Doubling times for all the high HER2 expressor cell lines ranged from 17.4 to 19.3 h, whereas doubling times for the low HER2 expressor cell lines ranged from 17.1 to 45 h.

### T-DM1 causing cell cycle arrest and apoptosis

**Figure 5:**
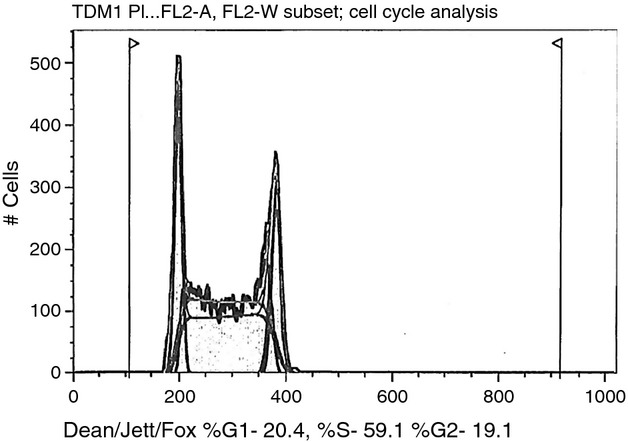
Cell cycle figure showing the absence of G_2_/M phase arrest in epidermal growth factor receptor-2 (HER2) nonoverexpressing cell line after treatment with T-DM1. The majority of the cells are in the S phase as shown in figure with the minority of the cells in the G_2_/M phase.

Next, we evaluated by cell cycle analysis whether T-DM1 may induce G_2_/M phase arrest and apoptosis in high HER2-expressing cells. In multiple experiments, we found the percentage of cells in G_2_/M phase to shift from a mean of 21.85 ± 1.89% in the pretreatment HER2 high expressor cell lines to a mean of 51 ± 8.9% (*P* = 0.01) after 48–72 h of exposure to 2 *μ*g/mL of T-DM1. T-DM1 showed activity (i.e., induction of G_2_/M phase arrest and apoptosis) against all USC cell lines tested including cell lines endowed with primary resistance to the cytostatic effects of T. In contrast, no significant G_2_/M phase arrest was noted in low expressor HER2 cell lines after exposure to T-DM1 (Fig.5).

### Effect of T-DM1 on USC xenograft

**Figure 6:**
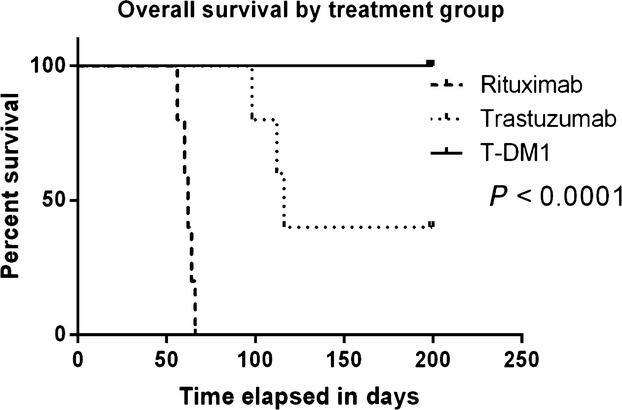
Overall survival in USC inoculated mice after treatment with weekly rituximab, T or T-DM1. Significantly prolonged overall survival is seen in the T-DM1-treated group compared to the other two treatment groups (*P* = 0.0001).

As described in the Methods section, drug treatment with rituximab (control antibody), trastuzumab, or T-DM1 intraperitoneally (i.p.) once per week was initiated 7 days after USC suspension inoculation in mice in each treatment group. All drug treatments were given weekly for 5 weeks after which mice were placed into observation until death or meeting criteria for euthanasia according to the IACUC guidelines. Palpable tumors and/or ascites were noted after approximately 3 weeks after USC tumor cell injection in the rituximab and trastuzumab groups. Specifically, all mice in the rituximab group (*n =* 5) had to be euthanized or died from tumor burden between days 56 and 66 after tumor injection, whereas three of five mice in the trastuzumab group required euthanasia or died from tumor burden between days 98 and 116 after tumor suspension injection (Fig.5). In contrast, all animals treated with T-DM-1 (i.e., five of five) remained free of detectable tumor for the entire duration of the study period (i.e., over 200 days, log rank *P* < 0.0001, Fig.6).

## Discussion

Our group has recently used whole-exome sequencing to analyze the genetic landscape of a large number of USCs 11. In this report, we found somatic copy number variations (CNV) to play a major role in the pathogenesis of USC, one that is likely at least as important as somatic point mutations (SNV). Consistent with this view, CNV analyses identified amplification of the *c-erbB-2* gene in 44% of the whole-exome-sequenced USC as well as major alterations in copy number in multiple additional genes involved in the NuRD and cell cycle pathways 11. The results of this comprehensive study confirmed the common amplification of the *C-erbB-2* oncogene previously reported in USC using different assays 7,12,13. These data suggest that either small molecule TK inhibitors targeting the HER2 intracellular pathway or antibodies targeting the extracellular portion of the HER2 receptor (i.e., trastuzumab, T-DM1) may represent innovative, potentially effective therapies against USC refractory to standard treatment modalities 1,14–16.

The identification of USC overexpressing HER2 at 3+ level by IHC and/or harboring amplification of the *c-erbB-2* gene by FISH may potentially represent the most effective way to guide selection of USC patients who may benefit more for T and T-DM1 therapy. However, demonstration of the contribution of the HER2/neu protein expression and *c-erbB-*2 gene amplification to the sensitivity to a specific targeted agent such as T-DM1 requires functional validation. In this study, we analyzed the presence of *c-erbB-2* gene amplifications and HER2 protein expression in a large series of primary, highly purified USC cell lines. To our knowledge, this is the first report investigating the activity of T-DM1 against HER2 overexpressing USC, a rare but highly aggressive variant of endometrial cancer. Our experiments consistently demonstrate that T-DM1 has a strong growth inhibitory effect on HER2-positive USC cell lines in vitro as well as in vivo in SCID mice harboring USC xenografts. While both T-DM1 and trastuzumab evoked similar ADCC against HER2 overexpressing USC cell lines, T-DM1 was found to be dramatically more effective than T in inhibiting cell proliferation and in inducing apoptosis after G_2_/M phase cell cycle arrest. Furthermore, while the majority of primary USC cell lines overexpressing HER2 were found to be resistant to the cytostatic effect of T, they remained highly sensitive to the exposure to T-DM1 in vitro and in vivo. The data presented in which USC cell lines develop or show primary resistance to T and retain sensitivity to TDM-1 are in agreement with results related to breast and gastric cancers overexpressing HER2 1,17–19. These results could have important clinical implication in current trials including an ongoing randomized phase II study of carboplatin/paclitaxel with or without T in patients with advanced or recurrent USC overexpressing HER2 (NCT01367002).

T-DM1 is the first antibody–drug conjugate receiving United States Food and Drug Administration approval for HER2-positive metastatic breast cancer. FDA approval followed the publication of the positive results from the randomized phase III trial of T-DM1 versus lapatinib plus capecitabine in patients previously treated with a taxane and trastuzumab (EMILIA).

Overall T-DM1 appears to be well tolerated as a single agent given at 3.6 mg/kg every 3 weeks. This dosing differs from the 6 mg/kg every 3-week trastuzumab dosing. TDM-1 also has a shorter half-life than trastuzumab (∼4 days for conjugated T-DM1 vs. 3–4 weeks for trastuzumab), which may also result in a decrease in adverse effects including cardiotoxicity associated with trastuzumab 18.

Despite the overexpression of HER2 in up to 61% of USC, 38% of clear cell, and 11% of grade 3 endometrioid adenocarcinoma of the uterus found in large cooperative Gynecologic Oncology Group studies (i.e., GOG 177 and GOG 181B) 20,21 as well as encouraging case reports on a limited number of patients 22–24, single agent, trastuzumab 4 mg/kg in week 1 then 2 mg/kg weekly until disease progression in stage III/IV or recurrent endometrial cancers at the phase II level failed to demonstrate significant activity (GOG-181B) 21. While the results of this study have been recently challenged due to the shortcomings in the design of GOG-181B study 25, these negative clinical findings do suggest that a significant number of endometrial cancer patients may potentially harbor disease endowed with primary resistance to T. Our current results seem to support this hypothesis because four of five of the *c-erbB2* amplified primary USC cell lines evaluated in this study were indeed found resistant to the cytostatic effect of T in vitro. While the mechanisms resulting in T resistance are not completely understood, recent studies have identified multiple potential molecular alterations to explain in vivo lack of clinical activity. Potential mechanisms include significant intratumoral heterogeneity in HER2 protein expression in USC 26, frequent phosphatidylinositol 3-kinase (PI3K) mutations or amplifications, as well as AKT and S6K phosphorylation in T-resistant tumors 27,28. Notably, in this study, two of five USC cell lines with *c-erbB2* amplification also harbored PIK3CA mutations.

Regardless of the mechanism of resistance to T active in USC, our experimental data clearly demonstrate that T-resistant USC may be highly sensitive to T-DM1 effects in vitro and in vivo. These results strongly support the working hypothesis that T-DM1 may represent a significantly more effective therapeutic tool when compared to T for the treatment of chemotherapy resistant/recurrent endometrial cancer overexpressing HER2.

In conclusion, we have shown that T-DM1 and T are similarly effective in inducing strong ADCC against primary *c-erbB-2* amplified USC cell lines in the presence of PBL. In vitro data also suggest that T-DM1 is dramatically more effective than T in inhibiting cell proliferation and in inducing apoptosis after G_2_/M phase cell cycle arrest. Our In vitro findings were confirmed in an in vivo model*,* where T-DM1 was significantly more effective at inhibiting HER2-positive USC xenograft growth and establishment than T. Due to the significant fraction of USC patients overexpressing HER2 7,13,29, T-DM1 may represent a treatment option for HER2-positive USC patients harboring disease refractory to chemotherapy and/or unresponsive to T.

## Conflict of Interest

The authors declare no conflict of interest or previous publication and fulfill all conditions required for authorship.
